# Caregiver Burden in Early Intervention: A Fuzzy-Set Qualitative Comparative Analysis of Causal Configurations

**DOI:** 10.3390/healthcare13233026

**Published:** 2025-11-24

**Authors:** Pau García-Grau, Julia Argente-Tormo, Gabriel Martínez-Rico, Rómulo J. González-García

**Affiliations:** Campus Capacitas, Catholic University of Valencia “San Vicente Martir”, 46001 Valencia, Spain; pau.garcia@ucv.es (P.G.-G.); julia.argente@ucv.es (J.A.-T.)

**Keywords:** caregiver burden, early intervention, psychosocial resources, fsQCA, family-centered practices

## Abstract

**Background/Objectives:** Caregiver burden is a well-documented phenomenon among families of children with disabilities, particularly within early childhood intervention contexts. Although family-centered practices aim to empower parents and foster collaborative relationships with professionals, the specific contribution of families’ psychological and social resources to caregiver burden remains insufficiently understood. This study examined the combinations of psychosocial conditions associated with both high and low levels of caregiver burden in families receiving early intervention services. **Methods:** A total of 117 families of children aged 0–6 years enrolled in an early intervention center in Valencia, Spain, participated in the study. Caregiver burden was assessed using the Zarit Burden Interview. Fuzzy-set qualitative comparative analysis (fsQCA) was applied to identify the combinations of conditions that were necessary and sufficient for the presence or absence of caregiver burden. **Results:** No single condition was necessary for the outcome, but several sufficient combinations were identified. High caregiver burden was associated with configurations involving low resilience, limited perceived social support, and reduced coping capacity, whereas low burden emerged from configurations characterized by stronger psychosocial resources, particularly high family confidence, resilience, and social support. The solutions showed high consistency and coverage, indicating robust explanatory models for both outcomes. **Conclusions:** These findings demonstrate that caregiver burden in early intervention arises not from isolated factors but from specific interactions among psychosocial conditions. Understanding these causal combinations provides a more nuanced perspective on family functioning and highlights the importance of strengthening resilience and social support within early intervention programs to reduce caregiver burden and promote family well-being.

## 1. Introduction

The term “caregiver burden” refers to the physical, psychological, social, and financial difficulties experienced by family members providing care [1,2]. This burden results from caregivers’ cognitive appraisal of stressors, influencing their well-being, social functioning, and health [3]. In Early Intervention (EI), burden is particularly relevant because families face prolonged and complex care demands during critical developmental periods, with substantial implications for family quality of life [4].

Parenting a child with disabilities involves multiple responsibilities and varies in satisfaction depending on contextual factors [5]. These responsibilities, often intensified in neurodevelopmental conditions, can generate significant physical, emotional, and financial strain, contributing to parental burden [6]. Although burden has been linked to child-related factors such as age and perceived severity, evidence shows that caregivers’ adjustment depends largely on their own resources and skills [7], making empowerment, confidence, and parental competence key factors in managing caregiving demands [8].

### 1.1. Contextualization of EI Services

The implementation of family-centered practices in EI has been a priority for support services internationally, reflecting a shift from expert-model, deficit-based approaches toward models that highlight environmental and family factors in child development [9,10,11]. The recommended practices promoted in the United States by the Division for Early Childhood (DEC) contributed to expanding this paradigm globally [12]. In Spain, EI has also evolved following these international influences, although the implementation of family-centered practices is still not fully achieved across services [8,13].

### 1.2. Capacity-Building Approach to Early Intervention Services

The promotion of child and family functioning is a central priority in EI services [14,15], which requires active collaboration with families throughout assessment, planning, and intervention. Dunst et al., [16] conceptualized help-giving practices as comprising relational and participatory dimensions, both of which have demonstrated positive effects on child and family outcomes [17]. Relational practices refer to respectful, empathetic interactions that recognize family strengths, whereas participatory practices involve shared decision-making and responsiveness to family priorities [17].

Consistent with this evidence, current EI frameworks emphasize strengths-based, collaborative approaches that build family capacity [12], with professionals supporting caregivers in enhancing everyday parenting opportunities and reinforcing parental self-efficacy [18]. Within this paradigm, capacity-building and empowerment are viewed as core outcomes of effective family-centered support [19].

### 1.3. Family Confidence in EI

Family confidence is one of the consequences of capacity-building and family-centered support services in EI. Family confidence is a component of a person’s self-efficacy beliefs [20] that refers to the ability to perform a task competently (for example, the family member’s confidence in helping a child participate in a home routine). Therefore, capacity-building practices in EI focus on partnering with caregivers by offering relevant information, guidance, and encouragement, while also fostering the development of their knowledge and skills to address both the child’s and the family’s needs in a specific context [20]. This approach contributes to improving the caregivers’ sense of competence and confidence in their parental role [10].

Previous studies have found that family confidence is a key outcome in attenuating the negative impact that caregiver burden has on family quality of life. In addition, family confidence has also demonstrated to strongly predict better perceptions of family quality of life [4] and decreased parenting burden [21] in the EI field. Furthermore, family confidence can also be understood at the family-system level, encompassing the family’s belief in its own ability to manage daily functioning and relational dynamics [22], rather than being limited to confidence in supporting the child’s development.

Ref. [13] found that increased confidence contributed to improving child functioning and family quality of life. Families that develop greater confidence in their capabilities are those that more effectively implement intervention strategies in daily life, which translates into greater adherence to the intervention plan and more consistent application of recommended strategies. This effective implementation generates a positive cycle where the child’s progress reinforces parental confidence, which in turn facilitates more successful intervention and reduces the perception of family burden.

### 1.4. Family Confidence and Caregiver Burden in EI

Family-centered EI practices prove to be an effective way to help cope with the perception of burden [9]. These practices aim to promote empowerment, collaboration, and shared decision-making by engaging families as active partners in their child’s development through participatory and relational practices [23,24,25]. Evidence shows that providing support in the form of opportunities for the family to acquire competencies, in turn, has an impact on their children and this affects their parental self-efficacy [9]. Furthermore, in the study by [26], with families of children with ASD, parents obtained a greater sense of parental self-efficacy when they felt more involved in their children’s intervention and were more satisfied with the training they received as part of these interventions, which contributes to reducing stress levels associated with burden.

### 1.5. The Configurational Perspective: fsQCA as a Methodology

Traditionally, research on family burden has employed linear approaches that seek to identify individual predictors through regression analysis. However, these methods may not adequately capture the inherent complexity of family phenomena, where multiple factors interact in non-linear ways to produce diverse outcomes. Qualitative comparative analysis (QCA) based on fuzzy sets (fsQCA) offers an alternative methodological perspective that recognizes equifinality—multiple paths can lead to the same outcome—and configurational causality—outcomes emerge from specific combinations of conditions rather than from independent additive effects [27].

This methodology is particularly appropriate for the study of family burden because it allows for the identification of complex causal configurations, recognizing that different combinations of factors can produce high or low levels of burden. Furthermore, fsQCA is well suited for studying complex social realities because it shows that there can be different combinations of factors leading to the same outcome, and that the absence of the outcome may result from entirely different circumstances [27]. For example, in EI, high family quality of life may emerge from combinations such as high family empowerment and strong social support, while low family quality of life may arise from different configurations, such as low resilience despite high social support.

In the EI context, where families present diversity of resources, needs, and characteristics, this methodological approach can provide more precise and applicable insights for the design of personalized interventions.

Although previous studies have examined the relationships between family confidence, child functioning, and caregiver burden, most have relied on linear approaches that do not capture how these factors operate jointly in Early Intervention. As a result, little is known about the specific combinations of psychosocial and service-related conditions that lead to high or low burden. This gap is particularly relevant in the Spanish EI context, where the implementation of family-centered practices remains uneven. To date, no configurational analyses have explored these interactions, limiting our ability to understand the complexity of caregiver experiences and to tailor supports accordingly.

Given the potential of configurational analysis and the aforementioned influence of family confidence and child functioning on caregiver burden in EI, the present study aimed to: describe the patterns of family confidence and caregiver burden in families receiving EI services in Spain; analyze the relationship between family confidence and caregiver burden scores; and identify the combinations of causal conditions that lead to both high and low levels of burden.

## 2. Materials and Methods

### 2.1. Participants

The study included 117 families of children aged 0–6 years receiving Early Intervention (EI) services across four Spanish autonomous communities.

Participating children were predominantly male (*n* = 42; 35.9%) and had a mean age of 43 months. The concept of family in this study refers to the primary caregiving unit responsible for the daily upbringing and participation of the child in EI activities, which may include biological parents, grandparents, or other legal guardians.

Primary respondents were mainly mothers (58.6%), followed by fathers (17.2%) and grandmothers (3.4%), while 19% of the questionnaires were jointly completed by both parents. Among individual respondents, mothers represented 75.9% (*n* = 44) and fathers 24.1% (*n* = 14) of the sample. The mean age of adult respondents was 36.5 years. The duration of EI service receipt ranged from 1 to 48 months (M = 16.6; SD = 12.5).

Regarding caregiving involvement, parents reported the time spent with their children on a 5-point scale (1 = very little to 5 = most of the time), with mothers averaging 4.24 and fathers 3.26. Caregivers rated the perceived severity of their child’s disability (M = 1.48; SD = 1.17) and the level of child’s functioning level (M = 3.64; SD = 0.88), indicating overall mild to moderate needs within the sample.

The sample size (*n* = 117) is adequate for fsQCA analyses. Methodological guidelines indicate that fsQCA is particularly suitable for small-to-medium samples, typically ranging from around 50 to 150 cases, as this range provides sufficient empirical diversity to construct meaningful truth tables and identify stable configurational patterns [27,28]. Previous studies in social and health sciences have employed similar sample sizes with robust results, supporting the adequacy of the current dataset for configurational analysis.

### 2.2. Instruments

Family Quality of Life and Caregiver Burden. Family outcomes were assessed using the Families in Early Intervention Quality of Life Scale (FEIQoL), validated and revised in Spain [29], The scale comprises 39 items rated on a 5-point Likert scale (1 = poor to 5 = excellent) across three dimensions: *Family Relations*, *Access to Information and Services*, and *Child Functioning*. For this study, the global Family Quality of Life index was used, demonstrating excellent internal consistency (α = 0.98).

Caregiver burden was evaluated using the 12-item Spanish adaptation of the Zarit Burden Interview (ZBI) [30,31]. Although originally designed for caregivers of dependent adults, this reduced version has been widely validated in family caregiving contexts, including parents of children with developmental disabilities. This tool has been widely used and numerous adaptations have emerged in a multitude of fields because it can be easily adapted for different populations. We used the reduced 12-item version because of its length and high internal consistency (α = 0.92) in Spanish [32]. In addition to the applicability of the items to caregivers receiving ECI services in Spain was a key for the instrument selection. Items are rated on a 5-point Likert scale (1 = never to 5 = always), and the overall score in this study demonstrated good reliability (α = 0.87). Its brevity and strong psychometric properties make it suitable for Early Intervention contexts, where participant time is limited.

The Family Confidence in Helping with Child and Family Functioning (Con-Fam) scale [33] was employed to measure parental self-efficacy across two domains: (1) *confidence in supporting the child’s participation and independence in daily routines* (20 items), and (2) *confidence in addressing family functioning aspects* such as informational, emotional, and material support needs (18 items). Responses are scored on a 4-point Likert scale (1 = “I am not quite sure how to help” to 4 = “I have complete confidence”). Reliability was excellent (α = 0.97 for child confidence; α = 0.93 for family confidence). The instrument demonstrated excellent reliability in the present sample (α = 0.97 for child confidence; α = 0.93 for family confidence), consistent with previous Spanish studies [13].

Information about the family and the child was collected through an online questionnaire using the same link. Families provided details such as their relationship to the child, caregiver’s age, child’s age and gender, and the amount of time each adult in the household spent with the child. The child’s level of difficulty or severity was assessed through a functional level scale ranging from 1 to 5 (1 = very low functioning, 5 = high functioning), which allowed for a more inclusive description of children with diverse diagnoses and developmental conditions.

Rationale for Instrument Selection. All instruments were selected for their empirical validation in the Spanish population and relevance to Early Intervention practice. The FEIQoL provides an EI-specific measure of family well-being, the Con-Fam captures the multidimensional construct of family confidence, and the abbreviated ZBI allows for assessing caregiver burden efficiently while maintaining robust psychometric quality [34,35].

### 2.3. Procedure

This study was conducted as part of a larger research project examining caregiver burden in Early Intervention, with institutional IRB approval obtained prior to data collection. The broader project explores multiple dimensions of family experiences, including parental confidence, gender-related differences in burden perceptions, and family quality of life outcomes. The present analysis focuses specifically on the subset of participants for whom complete data on family quality of life and caregiver burden were available.

Data collection was facilitated through electronic surveys distributed by EI professionals and service providers to families enrolled in their programs. The survey integrated all measurement instruments along with sociodemographic questions into a single digital platform. Participation was entirely voluntary and confidential, with families accessing the questionnaires via a secure electronic link. Prior to survey completion, participants were required to review and provide consent through a mandatory informed consent statement embedded within the platform. Eligibility criteria included: (a) being a primary caregiver of a child aged 0–6 years receiving EI services; (b) having at least six months of service participation; and (c) providing informed consent. Families were excluded if the child had an acute medical condition or if the caregiver declined to participate. Given the distribution method through multiple service providers, the total number of families who received invitations could not be determined, precluding calculation of an exact response rate. For inclusion in the final analytical sample, cases were retained only if participants had completed at least 90% of scale items, ensuring data quality and reliability for subsequent analyses.

All participating EI centers across the four autonomous communities followed a shared professional framework. Specifically, these centers implement the DEC Recommended Practices and are integrated within the organizational network Plena Inclusión España, which provides common guidelines, professional development, and quality standards for service delivery. This shared framework contributes to a substantial degree of uniformity in the sampling frame across regions, despite potential administrative differences among autonomous communities.

The study was approved by the Research Ethics Committee of Catholic University of Valencia (Approval Code: UCV/2018-2019/111), adhering to the principles of the Declaration of Helsinki. Electronic informed consent was obtained before participation, and confidentiality was guaranteed.

### 2.4. Data Analysis

Data were analyzed using fuzzy-set Qualitative Comparative Analysis (fsQCA), an approach suited to exploring how different combinations of conditions (i.e., psychosocial and contextual factors) jointly explain an outcome through the principle of equifinality. All analyses were performed using fsQCA software, version 4.1, following standard procedures for calibration, truth table construction, and solution derivation. Descriptive statistics (means, standard deviations, and correlations) were first computed to characterize the sample and contextualize the fuzzy-set calibration. Cases with more than 10% missing data were excluded; the remainder were analyzed using pairwise deletion, as missingness was random and minimal. Scale reliability was verified through both Cronbach’s alpha and rho_A coefficients [36]. All conditions were calibrated into fuzzy sets (0 = full non-membership, 1 = full membership) using the 10th, 50th, and 90th percentiles as empirical anchors [37], following methodological recommendations for fsQCA [38]. Necessity analyses identified conditions systematically present or absent among cases with the outcome, using a consistency threshold of 0.90 to define strict necessity. Sufficiency analyses were performed using truth tables, applying a frequency cutoff of 1 and a minimum consistency threshold of 0.80. Three types of solutions (complex, parsimonious, and intermediate) were generated, with the intermediate solution selected for interpretation due to its balance between empirical robustness and theoretical coherence [27].

## 3. Results

To carry out the analysis using Fuzzy-set Qualitative Comparative Analysis (fsQCA), a descriptive analysis was initially performed and the corresponding calibration values were calculated (Table 1). The descriptive statistics show a sample of 117 participants with mean scores ranging from 0.95 for the number of sessions to 3.64 for the child’s functional level. The variability observed across all variables provides an adequate basis for calibration into fuzzy sets, using the 10th, 50th, and 90th percentiles as anchor points to establish the thresholds for low, intermediate, and high membership to each condition.

### 3.1. Analysis of Necessary Conditions

The analysis of necessary conditions (Table 2) to explain both the presence and absence of burden shows that none of the evaluated conditions reaches the consistency threshold of 0.90 established by [39] as the criterion for considering a condition as necessary. The highest consistency values are observed for the child’s low functioning level (~Nfuc = 0.738) and the low levels of number of sessions (~Sesc = 0.765) in explaining low levels of burden, although neither reaches the critical threshold. These values indicate that none of the conditions alone is systematically common among cases with burden, which reinforces the need to analyze causal combinations through sufficiency analysis and confirms the configurational nature of the phenomenon under study.

### 3.2. Analysis of Sufficient Conditions

The sufficiency analysis identified combinations of causal conditions that lead to both high and low levels of caregiver burden. In constructing the truth table, a minimum consistency threshold of 0.80 was established, following a natural break in the distribution of consistency scores [39]. High levels of burden are explained by two sufficient configurations.

Regarding high levels of burden, two sufficient configurations were identified. In both, low family confidence (Cafc) emerges as a core and recurrent condition, highlighting its pivotal role in shaping caregivers’ perceptions of burden. The first configuration (H1: ~Sesc and ~Cafc) combines few therapeutic sessions with low family confidence. This configuration (Raw coverage = 62.7%, Unique coverage = 12.6%, Consistency = 80.8%) shows that limited therapeutic contact, together with families’ lack of perceived confidence, substantially contributes to increased levels of burden. Here, raw coverage represents the proportion of cases with high burden that are explained by this specific configuration, whereas unique coverage indicates the proportion explained exclusively by it, without overlapping with other configurations. Consistency, in turn, reflects the degree to which the configuration is a reliable subset of the outcome, that is, the extent to which the combination consistently leads to high burden. The second configuration (H2: ~Nfuc and ~Cafc) associates low child functional level with low family confidence (Raw coverage = 60.3%, Unique coverage = 10.2%, Consistency = 78.5%). This pathway suggests that when children exhibit reduced functional abilities and caregivers simultaneously feel unconfident in managing everyday challenges, the experience of burden intensifies. Taken together, both configurations achieve a solution coverage of 72.9% and a solution consistency of 78.0%, indicating that they jointly explain nearly three-quarters of the cases characterized by high burden, with a coherent and robust pattern of sufficiency.

In contrast, low levels of burden are explained by three consistent and theoretically meaningful configurations. Across these, family confidence with helping the family (Cafc) and family confidence with helping the child (Canc) appear as protective factors that mitigate caregivers’ perceived burden, while functional level (Nfuc) and number of sessions (Sesc) act as contingent elements whose effects depend on their combination with the other conditions. The first configuration (L1: ~Nfuc and Cafc) (Raw coverage = 56.2%, Unique coverage = 13.7%, Consistency = 83.1%) shows that even when children have lower functioning levels, high family confidence compensates for this limitation, allowing caregivers to handle daily demands with less strain. The second configuration (L2: ~Nfuc and Sesc and Canc), showing a Raw coverage = 39.4%, Unique coverage = 3.9%, and Consistency = 83.2%, indicates that, under conditions of low child functioning, frequent therapeutic sessions combined with high confidence in helping the child act as a buffer against burden, reflecting the benefits of engagement and perceived self-efficacy. The third configuration (L3: ~Sesc and Cafc and Canc) (Raw coverage = 49.2%, Unique coverage = 8.4%, Consistency = 82.6%) suggests that fewer sessions do not necessarily lead to burden when both family and child confidence are high, implying that empowered and self-reliant family dynamics can offset the need for intensive professional support. Overall, these three configurations yield a solution coverage of 76.9% and a solution consistency of 79.9%, confirming that they jointly account for more than two-thirds of the cases with low levels of burden and display a high level of empirical reliability. A detailed summary of these configurations and their statistical indicators is presented in Table 3.

Taken together, these findings reinforce the pivotal role of family confidence (Cafc) as a decisive condition in both directions of the outcome. Its absence consistently amplifies burden, while its presence, particularly when combined with child confidence (Canc), acts as a protective mechanism that reduces burden. Moreover, number of sessions (Sesc) and functional level (Nfuc) operate as situational moderators: reduced functioning or fewer sessions heighten burden only in the absence of family confidence, whereas strong family confidence mitigates these effects. This pattern underscores the importance of family empowerment and perceived competence as key levers for reducing caregiver strain.

**Figure 1:**
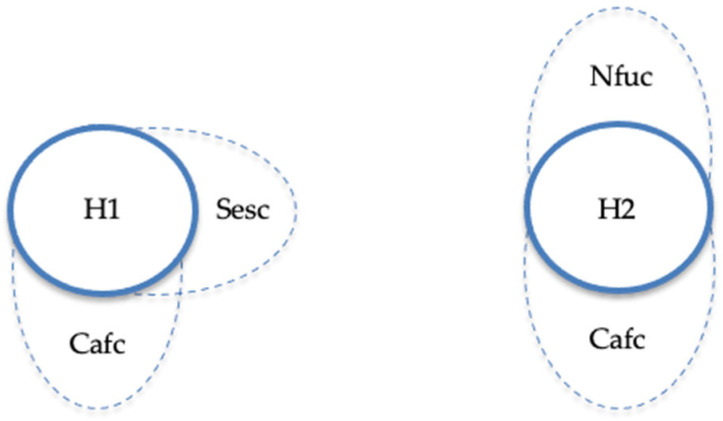
Causal configurations for high and low levels of burden. Note. Nfuc refers to functional level, Sesc = number of sessions, and Cafc = family confidence with helping the family. An ellipse outlined with a solid line signifies the presence of the condition, while an ellipse with a dotted line indicates its absence. If a condition is not pertinent to a given configuration, no ellipse is depicted.

**Figure 2:**
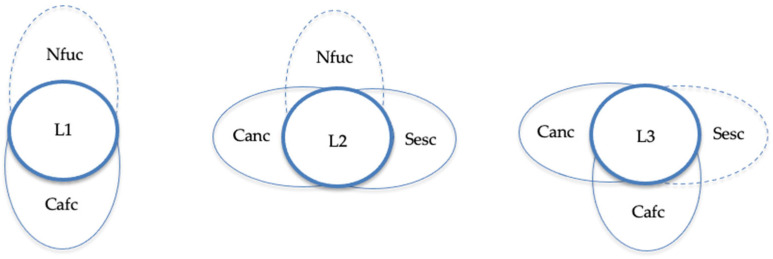
Causal configurations for low levels of burden. Note. Nfuc refers to functional level, Sesc to number of sessions, Cafc to family confidence, and Canc to child confidence. An ellipse outlined with a solid line signifies the presence of the condition, while an ellipse with a dotted line indicates its absence. If a condition is not pertinent to a given configuration, no ellipse is depicted.

These configurations are visually represented in Figure 1 and Figure 2, which illustrate the causal patterns associated with high and low levels of caregiver burden, respectively. Figure 1 illustrates the causal configurations leading to high levels of caregiver burden. In both solutions, the absence of family confidence (Cafc) is central, reinforcing its key role in explaining burden. Configuration H1 combines few therapeutic sessions (Sesc) with low family confidence, while H2 links low child functional level (Nfuc) and low family confidence. Together, they show that insufficient confidence within the family system, whether accompanied by limited sessions or low child functioning, consistently leads to higher burden.

Figure 2 shows the causal configurations leading to low levels of caregiver burden. Across the three solutions, the presence of family confidence (Cafc) and child confidence (Canc) acts as a protective factor that reduces perceived burden. Configuration L1 combines low child functional level (Nfuc) with high family confidence, L2 joins low functional level, high number of sessions (Sesc), and high child confidence, and L3 integrates high family and child confidence with few sessions. Together, these configurations illustrate that strong family confidence buffers burden, even when functional limitations or limited service use are present.

## 4. Discussion

The results obtained through fuzzy-set qualitative comparative analysis (fsQCA) provide solid evidence of the complex and configurational nature of family burden in the Early Intervention (EI) context. This methodological approach goes beyond traditional linear associations, exploring how different combinations of conditions converge toward high or low levels of burden. The application of fsQCA confirms that caregiver burden can rarely be explained by a single variable, reinforcing the importance of considering causal configurations and equifinality in applied social research, particularly in family intervention.

The analysis of necessary conditions revealed that none of the evaluated variables—functional level, number of sessions, family confidence, and child confidence—reached the 0.90 consistency threshold required to be considered necessary. This aligns with previous research showing that family burden is a multidimensional phenomenon and that isolated effects are insufficient to explain the outcome [21]. The absence of necessary conditions highlights a distinction from correlation-based approaches, which typically identify independent linear predictors. By contrast, the fsQCA logic emphasizes equifinality: multiple causal paths can lead to the same outcome, a principle especially relevant in family contexts where interactions among child characteristics, resources, and family dynamics produce heterogeneous outcomes.

The sufficiency analysis identified two consistent configurations explaining high levels of burden, both with low family confidence as the central condition. The first combines a low number of sessions with low family confidence, suggesting that limited professional support and a perception of low competence create conditions conducive to burden. The second combines low confidence with a low functional level of the child, indicating that significant child needs exacerbate burden when caregivers feel less self-efficacy. This is consistent with studies showing that child functioning alone does not directly cause burden, but that its impact is mediated by parental confidence [21].

These findings are strongly supported by the literature. The centrality of low parental confidence/self-efficacy as a core factor in burden is consistent with the theoretical model of [19], which posits that capacity-building practices in EI services enhance parental self-efficacy, reducing stress and improving family quality of life. A recent study [41] also found burden to be negatively associated with caregivers’ self-efficacy and quality-of-life dimensions. In addition, the first configuration corroborates findings in the Spanish context [13,29], where limited implementation of family-centered practices restricts participation and competence, intensifying perceived burden.

The analysis of configurations associated with low burden revealed three sufficient pathways, confirming greater diversity in protective strategies and reinforcing the principle of equifinality. The most prominent configuration combines low child functional level with high family confidence, underscoring the role of confidence as a protective factor even in contexts of functional dependence [4]. This supports empowerment-based approaches in EI [42]. The second configuration suggests that intervention frequency can reduce burden when needs are severe, while the third indicates that a low number of sessions does not necessarily increase burden if the family maintains high confidence—an outcome aligned with family-centered approaches promoting generalization and autonomy [13].

Configurations leading to low burden consistently include high levels of family or child confidence, reinforcing the idea that families who feel more competent implement intervention strategies more effectively [43]. This effective implementation generates a virtuous cycle: families adhere better to the plan, observe progress, and experience reduced burden [23]. A recent study in India [44] also showed that implementing family-centered practices improved caregiver strain and empowerment across contexts, with child severity being the only predictor of variation in benefit.

Some results diverge from previous studies that identified functional dependence as the main predictor of burden [45]. In our analysis, functional level appears only as part of specific configurations and never as a dominant condition. This discrepancy may reflect methodological differences: regression models assess net effects, whereas fsQCA shows that low functional level contributes to burden only when combined with additional unfavorable conditions. Our findings support the model of [9], where parental self-efficacy mediates the relationship between stressors and outcomes, and echo results showing that low confidence mediates the impact of stressors on Family Quality of Life [4]. Contextual factors may also contribute: families engaged with EI services may experience uniformly high caregiving demands, making psychological resources more explanatory than variations in daily care.

The results should be interpreted considering the heterogeneity of families receiving EI services. The identified configurations may vary depending on child characteristics, age at intervention onset, socioeconomic context, or cultural factors. Variables not included—such as informal support networks or differences in the implementation of family-centered practices—may also act as protective factors. Future research should incorporate measures of professional practices, community resources, and social support to refine the understanding of these configurations.

Our results also confirm a clear causal asymmetry between configurations leading to high versus low burden: the conditions explaining high levels are not simply the inverse of those predicting low levels. This finding, consistent with fsQCA principles, underscores the need for configurational approaches and suggests that preventing burden and reducing existing burden may require differentiated strategies tailored to each family’s situation.

Several mechanisms may explain how the identified configurations lead to different levels of burden. Family confidence may act as a cognitive mediator shaping caregivers’ appraisal of stressors, consistent with transactional stress theory. Confidence may facilitate more effective coping strategies, greater personal control, and better implementation of intervention plans. Although the literature is not unanimous regarding the primacy of psychological versus structural factors [46], our results suggest that the quality and empowerment focus of sessions may be more relevant than frequency [17].

Confidence in helping the child may also reflect perceptions of child progress, consistent with findings linking higher confidence to better perceptions of functioning and Family Quality of Life [13].

### 4.1. Implications for Future Practice and Research

We consider that our configurational analysis enriches and refines understanding of the phenomenon by positioning low family confidence not as the only factor, but as an indispensable catalyzing factor that, when interacting with other stressors, triggers the experience of burden. The configurations for low burden paint a hopeful and complex picture where there is no single recipe for family well-being, but rather a series of complementary strategies that EI services can implement: prioritizing empowerment as a cross-cutting element, adjusting session frequency according to needs, and working toward family autonomy with the goal that high confidence allows for a gradual reduction in service dependence without increasing burden.

These findings have direct implications for the design and delivery of EI services, highlighting the need for professionals to prioritize capacity-building practices that strengthen family confidence and competencies, going beyond merely providing direct therapy to the child [23,47]. There is no single solution, so services must evaluate each family’s specific configuration to offer individualized supports to increase their sense of competence. These results provide strong arguments for advocating a family-centered and strengths-based EI model, even in contexts where it is not yet fully implemented [48], as is the case in Spain.

### 4.2. Limitations

Among the study’s limitations are the sample size and the possible exclusion of other relevant variables such as formal and informal social support or financial stress. Future research should employ longitudinal designs to establish causality and observe the evolution of these configurations, include broader contextual variables to refine the models, and conduct in-depth qualitative studies to understand the subjective experience behind the identified configurations. This range of options reinforces the need for an individualized assessment of each family to identify which path toward burden reduction is most viable and appropriate, thus personalizing interventions in the most effective manner. Therefore, there is a need for EI services to assess and intervene in specific ways to strengthen parental self-efficacy, as this is the mechanism that can transform the way families perceive and manage caregiving demands, even when these are high.

## 5. Conclusions

In conclusion, this study uses an innovative configurational approach (fsQCA) to show that caregiver burden in Early Intervention is a multifaceted phenomenon shaped by the interaction of psychosocial and contextual factors rather than by the isolated effect of a single variable. The most consistent result is the central role of family confidence (Cafc): its absence characterizes pathways leading to high burden, while its presence underlies most protective configurations. This highlights empowerment and parental self-efficacy as core mechanisms that shape caregivers’ experiences.

The findings also illustrate the principle of equifinality, demonstrating that there is no single path to preventing or mitigating burden. Different combinations of child functional level, intervention frequency, and confidence can lead to similar outcomes, underscoring the need for flexible, family-tailored interventions instead of standardized procedures.

Overall, this study provides empirical support for advancing EI practices toward family-centered, strengths-based models. Prioritizing the enhancement of caregivers’ confidence and capabilities should be considered a central strategy for promoting child development and improving family quality of life.

## Figures and Tables

**Table 1:** Descriptive statistics and calibration values.

		Nfuc	Sesc	Cafc	Canc	Burden
N		117	117	117	117	117
M		3.64	0.95	2.88	2.86	3.22
SD		0.88	0.28	0.61	0.67	0.84
Minimal		1.00	0.5	1.17	1.00	1.47
Maximum		5.00	2.00	4.00	4.00	5.00
Calibration Values						
Percentile	10	3.00	0.53	2.09	2.00	1.27
	50	4.00	1.00	3.00	2.95	2.08
	90	5.00	2.00	3.61	3.70	3.25

Note: Nfuc refers to the child’s functioning level, Sesc to number of sessions, Cafc to confidence with helping the child, and Canc to confidence with helping the child. M = Mean; SD = Standard deviation.

**Table 2:** Analysis of necessary conditions for high and low burden levels.

	Burden			~Burden	
	Consistency	Coverage		Consistency	Coverage
Nfuc	0.492	0.649	~NFuc	0.738	0.596
Sesc	0.574	0.689	~Sesc	0. 765	0.646
Cafc	0.476	0.481	~Cafc	0. 495	0.490
Canc	0.581	0.581	~Canc	0. 589	0.588

Note: Nfuc refers to the child’s functioning level, Sesc to number of sessions, Cafc to confidence with helping the child, and Canc to confidence with helping the child. ~ Refers to low levels.

**Table 3:** Intermediate solution of the sufficiency analysis high and low levels of burden.

Group						Coverage		
	Path	Nfuc	Sesc	Cafc	Canc	Raw	Unique	Consistency
**High levels of burden**								
	H1		○	○		0.627	0.126	0.808
	H2	○		○		0.603	0.102	0.785
	Solution Coverage = 0.729; Solution Consistency = 0.780							
**Low Levels of burden**								
	L1	○		●		0.562	0.137	0.831
	L2	○	●		●	0.394	0.039	0.832
	L3		○	●	●	0.492	0.084	0.826
	Solution Coverage = 0.769; Solution Consistency = 0.799							

Note; ● = presence of condition, ○ = absence of condition. Expected Vector for high levels of overbuden 1.1.1.1. (1 presence); expected Vector for low levels of buden 0.0.0.0 (0: absence) using the [40]. Nfuc refers to child’s functioning level, Sesc = number of sessions, Cafc = family confidence with helping the family, and Canc = family confidence with helping the child.

## Data Availability

The original contributions presented in the study are included in the article, further inquiries can be directed to the corresponding authors.

## Abbreviation

The following abbreviation is used in this manuscript:

EI	Early Intervention
